# Squamous Cell Carcinoma—A Summary of Novel Advances in Pathogenesis and Therapies

**DOI:** 10.3390/cancers14102523

**Published:** 2022-05-20

**Authors:** Imran Khan, Charbel Darido

**Affiliations:** 1 Peter MacCallum Cancer Centre, 305 Grattan St., Melbourne, VIC 3000, Australia; imran.i.khan@petermac.org; 2 The Sir Peter MacCallum Department of Oncology, The University of Melbourne, Parkville, VIC 3010, Australia

Squamous cell carcinomas (SCCs) are cancers of epithelial cells lining the aerodigestive and genitourinary tract [1]. SCCs are known to occur in the skin, head and neck, oesophagus, lung, cervix, pancreas, thyroid, bladder, and prostate [2]. SCCs are the leading causes of cancer worldwide [3], and their incidence is on the rise due to an increase in exposure to carcinogens such as tobacco, alcohol consumption, sunlight, and human papilloma virus (HPV) infection.

In this Special Issue, experts in the field describe the cellular and molecular events in SCC development, recurrence, and metastasis. They provide an updated snapshot on our understanding of the heterogeneity of SCC pathogenesis with novel opportunities in precision therapeutics to achieve better clinical outcomes. In addition, the researchers highlight the current advances in therapy development, outcome-predicting biomarkers, and molecular mechanisms of therapy resistance along with possible remedial measures. 

Bai et al. [4] describe the molecular mechanisms of oral epithelium (OE) formation, repair, and homeostasis. They focus on key molecular mechanisms involved in OE terminal differentiation that go awry during oral SCC development. The authors also describe current therapeutics available to treat oral SCC and propose targeted approaches to restore OE differentiation as a novel oral SCC treatment strategy.

Thai et al. [5] describe the risk factors and molecular mechanisms involved in the development of cutaneous SCC. They explain how chronic exposure to ultraviolet radiation or immunosuppressive and kinase inhibiting drugs used to treat other ailments are *bona fide* risk factors for the development of cutaneous SCC. In addition, they discuss the risk for people suffering from chronic lymphocytic leukaemia, Marjolijn’s ulcers, inherited bone marrow failure syndrome, and human papilloma virus (HPV) infection. The authors define common genetic aberrations involved in the development of cutaneous SCCs including the dysregulation of Notch signalling and inactivation of *TP53* and *CKN2A* tumour-suppressor genes. Finally, the authors describe current therapeutics available to treat cutaneous SCC including tumour-resection surgery and chemotherapy to treat localised cancers and the benefit of immunotherapy and kinase inhibitors in the treatment of advanced metastatic disease.

Garcia-Sancha et al. [6] describe mechanisms of immunotherapy resistance in cutaneous SCC. Similarly to Thai et al. [5], they highlight the immune-checkpoint inhibitors in cutaneous SCC treatment. In addition, the authors describe predictors of immunotherapy outcomes as well as primary and acquired mechanisms of immunotherapy resistance. Finally, the authors demonstrate how therapy can be improved by the combination of immunotherapy, radiotherapy and chemotherapies, immunotherapy with oncolytic viruses, cancer vaccines, and other therapies in treating cutaneous SCC.

SCC of the anus is a rare malignancy with an increasing incidence and mortality rates attributed to an aging population. Currently, chemoradiation therapy (CRT) is the standard of care treatment for early-stage disease; however, CRT involves a pronounced level of toxicity. It is not clear whether CRT is appropriate for early-stage anal SCC. Meill and Bazan [7] summarise the clinical trials and retrospective studies comparing radiation therapy (RT) to CRT, local excision versus CRT, and RT versus systemic treatment of early-stage anal SCC. In addition, the authors describe the utility of novel radiotherapy techniques to treat anal SCC. Overall, the authors propose investigations into the de-escalation of therapy to ameliorate therapy-induced toxicities and improve the prognosis in patients with early-stage anal SCC.

Oropharyngeal SCC incidence rates have been increasing. Oropharyngeal SCC is a highly heterogenous disease with distinct viral (HPV) and non-viral (alcohol, tobacco, etc.) aetiologies with differing clinical presentation, pathogenesis, and prognosis. HPV-positive oropharyngeal SCC have a higher propensity to metastasise to distant organs with frequent lymph-node involvement. Nevertheless, the prognosis of HPV-positive oropharyngeal SCC is generally better than non-viral oropharyngeal SCC. However, current therapeutics do not take the tumour HPV status into account. Bozec et al. [8] describe the impact of HPV status on oropharyngeal SCC treatment outcomes. The authors indicate that early-stage or locally advanced resectable HPV-positive oropharyngeal SCC can be treated with surgery or RT with favourable clinical outcomes. In contrast, at similar disease stages, the surgical interventions to treat non-viral oropharyngeal SCC lead to better clinical outcomes. The authors conclude that the overall surgical interventions lead to better oncologic outcomes; however, further consideration of the viral status needs to be incorporated in future therapeutic interventions.

Koo et al. [9] describe the mutational landscape of oral SCC with a special emphasis on non-smoking and non-drinking (NSND) patients. They used targeted gene-panel sequencing to identify frequent mutations in the tumour-suppressor gene *CDKN2A,* amplification of *EGFR*, and deletion of *BRCA2* in NSND oral SCC compared to smoking and drinking (SD) oral SCC patients. Overall, this study excluded HPV as a major driver of oral SCC development in NSND patients and identified critical differences in mutational landscapes of NSND and SD oral SCC patients driving oncogenic events.

Cathepsin S is a lysosomal cysteine protease and is upregulated in several human cancers including oral SCC [10]. Tu et al. [10] used a combination of animal and in vitro studies to identify Cathepsin-S-activated neuronal PAR_2_ (protease-activated receptor-2), which induces cancer pain in oral SCC patients. This research opens new avenues in cancer-related pain management in oral SCC.

Finally, Kessler et al. [11] describe a novel treatment of head-and-neck SCC with RAS mutations. The RAS family of proto-oncogenes (KRAS, NRAS, and HRAS), which regulates the PI3K signalling pathway, is highly mutated in SCC [11]. Hyperactivation of RAS has been observed in head-and-neck, lung, and urinary-tract SCC. The authors describe the utility of Tipifarnib, an inhibitor of HRAS activity as mono or combination therapy in head-and-neck SCC. Using patient-derived xenograft mouse models, the researchers demonstrated that Tipifarnib sensitises head-and-neck SCC tumours to cetuximab (EGFR inhibitor), cisplatin (chemotherapy), Palbociclib (CDK4 and CDK6 inhibitor), and INK-128 (mTOR inhibitor).

In summary, this Special Issue describes the recent advances in SCC development and summarises the state-of-the-art therapeutics to treat primary and metastatic SCC. As Guest Editor, I thank all of the authors for their contribution to the challenging landscape of SCC biology and treatment.
